# Development and Validation of a Tumor Mutation Burden-Related Immune Prognostic Signature for Ovarian Cancers

**DOI:** 10.3389/fgene.2021.688207

**Published:** 2022-01-11

**Authors:** Mengjing Cui, Qianqian Xia, Xing Zhang, Wenjing Yan, Dan Meng, Shuqian Xie, Siyuan Shen, Hua Jin, Shizhi Wang

**Affiliations:** ^1^ Key Laboratory of Environmental Medicine Engineering, Ministry of Education, School of Public Health, Southeast University, Nanjing, China; ^2^ Clinical Laboratory, Affiliated Tumor Hospital of Nantong University (Nantong Tumor Hospital), Nantong, China

**Keywords:** ovarian cancer, tumor mutation burden, immune risk score, prognostic biomarkers, immune checkpoint

## Abstract

Ovarian cancer (OC), one of the most common malignancies of the female reproductive system, is characterized by high incidence and poor prognosis. Tumor mutation burden (TMB), as an important biomarker that can represent the degree of tumor mutation, is emerging as a key indicator for predicting the efficacy of tumor immunotherapy. In our study, the gene expression profiles of OC were downloaded from TCGA and GEO databases. Subsequently, we analyzed the prognostic value of TMB in OC and found that a higher TMB score was significantly associated with a better prognosis (*p* = 0.004). According to the median score of TMB, 9 key TMB related immune prognostic genes were selected by LASSO regression for constructing a TMB associated immune risk score (TMB-IRS) signature, which can effectively predict the prognosis of OC patients (HR = 2.32, 95% CI = 1.68–3.32; AUC = 0.754). Interestingly, TMB-IRS is also closely related to the level of immune cell infiltration and immune checkpoint molecules (PD1, PD-L1, CTLA4, PD-L2) in OC. Furthermore, the nomogram combined with TMB-IRS and a variety of clinicopathological features can more comprehensively evaluate the prognosis of patients. In conclusion, we explored the relationship between TMB and prognosis and validated the TMB-IRS signature based on TMB score in an independent database (HR = 1.60, 95% CI = 1.13–2.27; AUC = 0.639), which may serve as a novel biomarker for predicting OC prognosis as well as possible therapeutic targets.

## Introduction

Ovarian cancer (OC) is one of the most common malignancies of the female reproductive system, with worldwide incidence second only to cervical cancer, ranking first in the number of deaths from female reproductive system-related tumors (Webb and Jordan, 2017). The low efficiency of early diagnosis and screening of OC is due to the location of ovaries deep in the pelvic cavity, nonpalpable body surface, and lack of typical symptoms at onset (Stewart et al., 2019). In addition, the tumor grows rapidly, and most patients already have disseminated lesions at the time of diagnosis (Orr and Edwards, 2018). First-line conventional treatments for OC are mainly surgery and chemotherapy (Wang et al., 2016; Wang et al., 2019). Since many OC patients exhibit primary or secondary resistance to chemotherapeutic agents, new therapeutic approaches need to be discovered to improve the prognosis of OC patients (Valmiki et al., 2021).

The tumor microenvironment (TME) plays an important role in tumor growth and therapy. As a critical part of the TME, immune cell infiltration can orchestrate innate and adaptive immune responses (Hinshaw and Shevde, 2019). With a deeper understanding of the tumor microenvironment, immunotherapy has been approved for the treatment of various types of advanced or recurrent cancers due to its long-term anti-tumor effects (Kruger et al., 2019). OC expresses highly immunogenic tissue-specific antigens, and immune infiltration is the main prognostic factor (Le Saux et al., 2020). Therefore, there is a strong biological basis for the development of immunotherapy for OC (Hao et al., 2018). Currently, checkpoint blockade is the most promising immunotherapy in OC (Ghisoni et al., 2019). However, the objective response rate of immunotherapy alone is not optimal (Wang et al., 2019). The combination of PD(L)-1 antibody and poly (ATP-ribose) polymerases (PARP) inhibitors or conventional chemotherapy has obtained a good response in clinical trials (Wang et al., 2019). Therefore, it is urgent to find molecular markers that can effectively predict the efficacy of OC immunotherapy and screen the appropriate immunotherapy population.

Tumor mutation burden (TMB) is defined as the total number of gene somatic mutations, base substitutions, gene insertion or deletion detected per million bases (Huo et al., 2020). TMB, as an important biomarker that can represent the degree of tumor mutation(Bi et al., 2020), is becoming an emerging biomarker that predicts prognosis and is sensitive to immune checkpoint inhibitors (ICIs) (Merino et al., 2020). Data from retrospective studies indicate that cancers with higher TMB are more likely to respond to ICIs(Snyder et al., 2014; Rizvi et al., 2015). For instance, Killock et al. found that higher TMB was significantly associated with improved survival in melanoma treated with programmed cell death protein 1 (PD-1) immune checkpoint blockade (Killock, 2020). Chalmers et al. reported that TMB could be accurately assessed using comprehensive genomic profiling (CGP) analysis, by which a large proportion of patients with high TMB across tumor types can benefit from immunotherapy (Chalmers et al., 2017).

However, the role of TMB associated immune genes in OC prognosis and the relationship between TMB associated immune genes and OC immune cell infiltration need further investigation. In the present study, somatic mutations and RNA-seq data of OC patients were obtained from TCGA. Subsequently, we analyzed the TMB prognostic value in OC and found that the higher TMB score group had a significantly better prognosis. According to the TMB grouping, 9 key TMB-related immune prognostic genes were selected out and used to construct a TMB-related immune risk score (TMB-IRS) signature that could effectively predict the outcome of ovarian cancer patients Finally, we explored the relationship between TMB and prognosis and validated the TMB-IRS signature based on TMB score in an independent database, which may serve as a novel biomarker and potential therapeutic target for predicting OC prognosis.

## Materials and Methods

### Data Collection and Preprocessing

A total of 436 patients with OC were collected from The Cancer Genome Atlas (TCGA) (https://portal.gdc.cancer.gov/) database, including somatic mutation, clinical information, survival information and gene-expression data (FPKM normalized). Based on the following inclusion criteria: 1) The patient’s pathological diagnosis is OC; 2) Complete mRNA expression profile; 3) Complete clinical information. Exclusion criteria: 1) Non-primary OC; 2) patients with missing mutation information and survival information; 3) patients who Relapsed OC. In all, we selected 271 OC samples as a training set, including corresponding clinical characteristics, such as age, cancer status, grade, stage, and race (Supplementary Table S1).

The gene names of all immune genes were downloaded directly from the website. From the Immunology database and Analysis Portal (ImmPort) database (https://immport.niaid.nih.gov) we downloaded the complete list of immune-related genes, including a total of 2483 immune-related genes (Supplementary Table S2).

### Calculation of TMB Scores and Prognostic Analysis

To evaluate the prognostic differences between different TMBs in OC patients, we performed the following analysis. In our study, the TMB score of each individual was calculated by, the number of mutations divided by exon length (30 MB). Then, OC samples were divided into high and low-TMB groups according to the median number. And further, Kaplan-Meier analysis was implemented for the comparison of differences in overall survival (OS) between the two groups. Visualization of the somatic mutation landscape of OC patients was done by using the “maptools” package in R. The version number of the R software used in this study is v 3.6.1.

### Differential Analysis

Based on TMB grouping, we first performed differential analysis to identify genes differentially expressed in the high- and low-TMB groups. Specifically, differentially expressed genes (DEGs) were obtained using the “limma” package in R. Among them, log2 | FC | > 0.58 (FC, fold change) and *p* < 0.05 are criteria. Visualization of DEGs was implemented by plotting volcano plots *via* the “ggplot2”, “Cairo” and “ggrepel” software packages in R.

### Construction and Validation of TMB-Related Immune Risk Score (TMB-IRS) Signature

TMB related immune prognostic genes in OC were screened out by stepwise analysis, as a way to construct a TMB-IRS signature that could effectively predict the prognosis of OC. Differential expression analysis was first performed to obtain TMB-associated genes. The above gene and immune gene sets were intersected so that differentially expressed immune genes were obtained. Further, genes with an expression level of 0 in more than 50% of the samples were removed from differentially expressed immune genes. Subsequently, Cox regression and LASSO regression were performed to obtain independent immune genes related to prognosis using the “glmnet” R package. Based on the corresponding regression coefficient *β* value, the risk score value of each sample was calculated by, TMB-IRS = *Σ* Cox coefficient of gene Xi × scale expression value of gene Xi.

Each sample was ranked according to the risk score and grouped by the median, and patients were therefore divided into low- and high-risk groups. The prognostic value of the signature was assessed by performing Kaplan-Meier (KM) analysis with a log-rank test, using the “survminer” R package. Using the “survival” ROC R software package, we plotted the receiver operating characteristic (ROC) curve over time to evaluate the accuracy of the signature.

Search the GEO database for OC cohorts with gene expression and prognostic information, and finally select the GSE26712 cohort as a reasonable validation set, *n* = 148. For comparability of data from different sources, gene expression from geo data were further log transformed.

### Relationship Between Clinicopathological Factors and TMB-IRS Signature

To evaluate whether the TMB-IRS could serve as an independent predictor of prognosis, we first employed univariate Cox regression analysis to look for clinical features associated with prognosis and then performed multivariate Cox regression analysis to look for independent factors. Besides, in order to comprehensively evaluate the prognosis of OC patients, we plan to establish a comprehensive assessment model that combines clinical information with the TMB-IRS signature. In brief, using the “rms” package in R, we constructed a nomogram that could predict 2-, 3-, and 5-years patient survival. To compare the consistency of the actual OS of OC with the predicted effect, calibration curves (2-, 3-, and 5-years survival prediction) were plotted, and the curve at 45 represented the nomogram with better prediction accuracy.

Further, we used the R survival package to calculate the concordance index (C-index) of TNM stage, TMB-IRS and nomogram for comparing the predictive ability of the three for the prognosis of OC patients. Meanwhile, decision curve analysis (DCA) at 2, 3 and 5 years were calculated to measure the clinical utility of our established nomogram. The *x*-axis represents the percentage of threshold probability, and the *y*-axis represents net income.

### Cibersort Database Analysis

In order to estimate the infiltration of immune cells, we used CIBERSORT online immune cell infiltration estimation analysis tool (http://cibersort.stanford.edu/). It is a tool to deconvolute immune cell subtype expression matrices based on linear support vector regression principles. In the present study, the tool was suitably employed to compare the proportions of 22 immune cells in the high- and low-TMB-IRS groups. The 22 types of immune cells included: 7 types of T cells (CD8^+^ T cells, naive CD4^+^ T cells, resting memory CD4^+^ T cells, activated memory CD4^+^ T cells, follicle-assisted T cells, regulatory T cells, and γδT cells), 3 types of B cells (naive B cells, memory B cells, and plasma cells) NK cells (resting NK cells and activated NK cells), and various myeloid cells (monocytes, M0 macrophages, M1 macrophages, M2 macrophages, resting dendritic cells, activated dendritic cells, resting mast cells, activated mast cells, eosinophils, and neutrophils). *p* less than 0.05 was set as the criterion for statistical significance.

### Statistical Analysis

The SPSS 20.0 was adopted for multivariate Cox regression analysis, with a probability of a stepwise entry of 0.05 and removal of 0.1. And the simple mathematical operation processes and all table making were completed by the software Excel. Univariate and multivariate Cox regression was carried out to analyze the relationship among gene expression, clinical features and prognosis. Additionally, the “survival ROC” package was used to plot the survival ROC in R (v 3.6.1). All analyses associated with prognosis were performed with the “survival” package. The probability threshold with a significant difference was set as *p* < 0.05.

## Results

### Landscape of the OC Mutation Profiles

**FIGURE 1:**
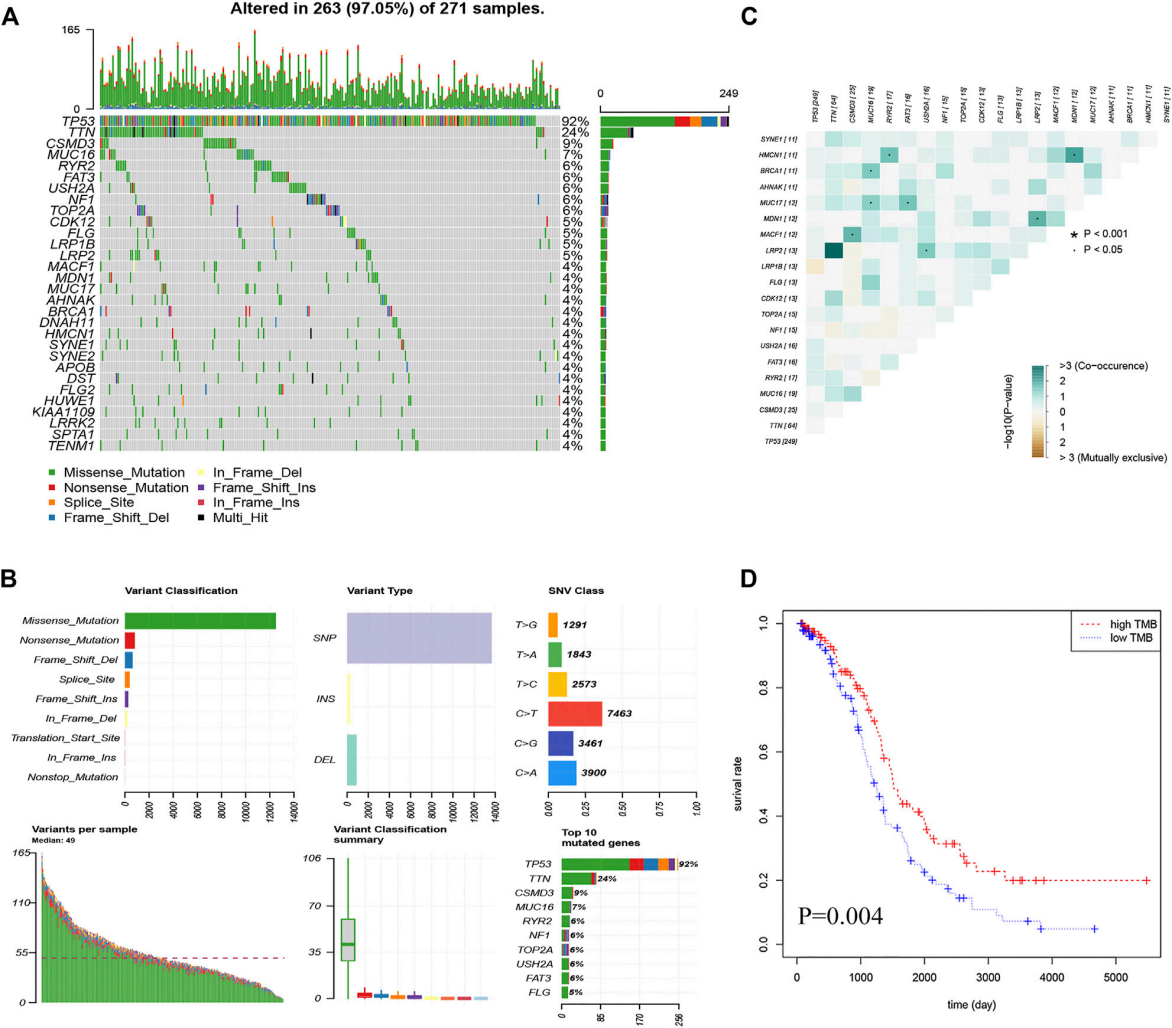
Landscape of the OC mutation profiles. **(A)** Waterfall plots present the landscape of the top 30 genes somatically mutated in 271 OC patients. **(B)** Various bar graphs show somatic mutations data. Variant classification reveal that missense mutations are the most; Variant type show the highest number of SNP (single nucleotide polymorphism); SNV(single nucleotide variants) class show the highest number of C > T; Variant per sample, with a median of 49; Summary of variant type; Top 10 mutated genes **(C)** Correlation Heatmap of the top 20 mutated genes **(D)** Kaplan-Meier curves show that OS in the high-TMB was significantly higher than in the low TMB group.

In total, we analyzed the somatic mutation profiles of 271 patients. As shown in Figure 1A, there were 263 samples with somatic mutation data, accounting for 97.05%. *TP53*, *TTN*, and *CSMD3* mutations are the top three mutated genes in OC samples, and *TP53* mutations are found in more than 92% of OC samples. Moreover, missense mutations were the most common mutation classification, single nucleotide polymorphisms (SNPs) showed a higher fraction in the variant type than insertion or deletion, and C > T was the most common single nucleotide variant (SNV) in OC (Figure 1B). Furthermore, the number of variants in each sample was calculated, and the mutation types were also shown in Figure 1B with different colors for OC. The co-occurrence and exclusive associations between mutated genes are shown in Figure 1C.

After calculating the TMB value of each sample (Supplementary Table S3), all patients were divided into high- and low-TMB groups according to the median and interquartile range [M(IRQ) = 1.947 (1.316, 2.684)]. Interestingly, patients in the low-TMB group have an obviously shorter OS than those in the high-TMB group with *p* = 0.004 (Figure 1D).

### Establishment and Evaluation of TMB-IRS Signature

**FIGURE 2:**
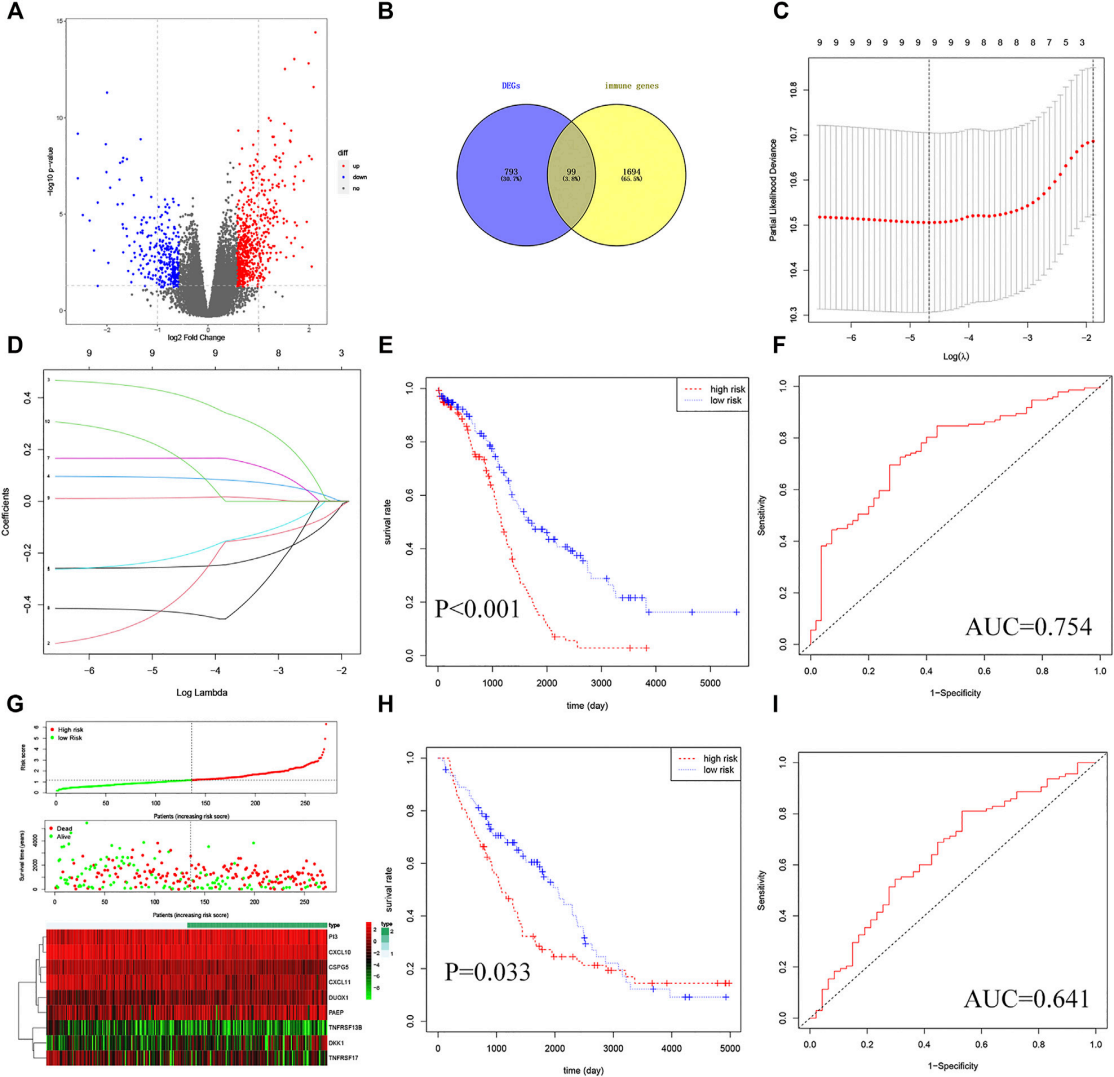
Establishment and validation of TMB-IRS signature. **(A)** Identification of 892 DEGs based on TMB score. **(B)** DEGs intersected with immune genes in a Venn diagram with 99 genes available. **(C)** Ten-time cross-validation for tuning parameter selection in the LASSO model. **(D)** LASSO coefficient profiles. **(E)** Kaplan-Meier curves show that OS was significantly different between the high- and low-risk groups in TCGA-OC. **(F)** The TMB-IRS signature is shown by the time-dependent ROC curve for predicting 2, 3, 5-years survival. **(G)** Risk score, survival status, and heatmap of 9 immune genes in patients with OC. **(H)** Kaplan-Meier curves show that OS in the low-risk was significantly higher than in the high-risk group in GSE26712. **(I)** Time-dependent ROC curve analysis of the TMB-IRS signature at 2, 3, 5 years in GSE26712.

To establish a TMB-IRS signature in the TCGA-OV cohort, multivariate Cox and LASSO analyses were employed to screen out independent immune genes related to prognosis. Specifically, a total of 892 differentially expressed genes were first differentially analyzed between the high- and low-TMB groups (Figure 2A, Supplementary Table S3). The DEGs above intersected with 1793 immune genes to obtain 99 differentially expressed immune genes (Figure 2B). Further, univariate Cox regression analysis obtained 12 immune genes related to disease prognosis (Supplementary Table S5). After eliminating two genes with 0 expressions in more than 50% samples, LASSO regression analysis was performed, resulting in 9 independent prognostic immune genes (Figures 2C,D), namely *CSPG5*, *CXCL10*, *CXCL11*, *DKK1*, *PI3*, *TNFRSF17*, *DUOX1*, *TNFRSF13B* and *PAEP*. Finally, based on the regression coefficients and gene expression of the above 9 genes, and TMB-IRS was calculated for each patient with the following formula:

$$\begin{array}{l} TMB-IRS=0.417*\exp DKK1+0.091*\exp PI3\\ \;\;\;\;\;\;\;+0.166*\exp DUOX1+0.013*\exp PAEP\\ \;\;\;\;\;\;\;+0.184*\exp CXCL10-0.254*\exp CSPG5\\ \;\;\;\;\;\;\;-0.392*\exp CXCL11-0.219*\exp TNFRSF17\\ \;\;\;\;\;\;\;-0.428*\exp TNFRSF13B \end{array}$$

Then, the risk score of each individual was calculated and ranked among OC patients, and then divided into high- and low-risk groups according to the median [M(IRQ) = 1.173 (0.798, 1.718)]. KM analysis indicated that patients in the high-risk group (*n* = 135) tended to have a worse prognosis compared to those in the low-risk group (*n* = 136) (Figure 2E, HR = 2.32, 95% CI = 1.68–3.32; *p* < 0.001). In addition, the survival ROC curve results showed that the TMB-IRS signature had relative accuracy in predicting the prognosis of OC (Figure 2F, 5-years AUC = 0.754). The risk curve and heatmap (Figure 2G) showed the patient risk score for each individual as well as the expression levels of the 9 genes.

### Validation of the TMB-IRS Signature

In order to verify the universal applicability of the TMB-IRS signature, the OC cohort downloaded in the GEO database was used as a validation set, and patients with missing mutation information and survival time less than 30 days were excluded. A total of 148 patients were analyzed for prognosis. According to the TMB-IRS formula established by the OC cohort in the TCGA database, the risk score of each patient in the validation set was calculated. According to the median TMB-IRS calculated by the TCGA database cohort, the validation set was divided into low-risk group and high-risk group. The results of KM analysis showed that TMB-IRS was significantly related to the prognosis (Figure 2H, HR = 1.46, 95% CI = 1.03–2.08; *p* = 0.033). The low-risk group had a better prognosis, while the high-risk group had a worse prognosis, which was consistent with the results of the TCGA database cohort. The ROC curve shows that the model has a good agreement between the predicted probability of OS and the actual probability (Figure 2I; 5-years AUC = 0.641).

### Correlations Between TMB-IRS and Clinical Variables

**FIGURE 3:**
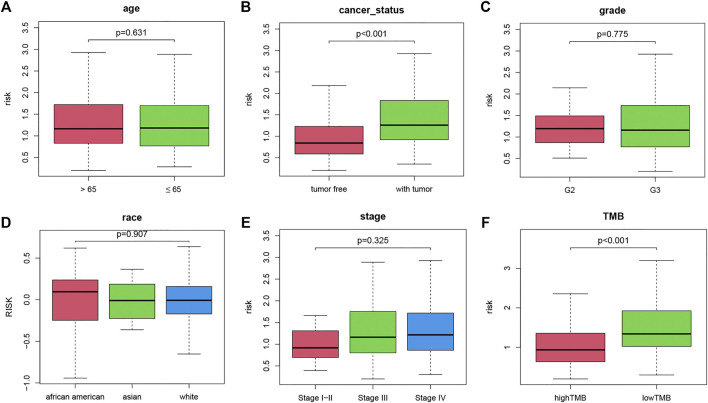
Boxplot of the relationship between clinicopathological factors and TMB-IRS. **(A–F)** Relationship between age, tumor status, grade, race, stage, TMB and TMB-IRS, respectively.

To investigate the correlation between clinical variables and the TMB-IRS, boxplots were drawn to visualize the immune risk profile across clinical subgroups. As shown in Figure 3, the immune risk score was significantly positively correlated with cancer status but negatively correlated with TMB. The risk score was significantly higher in the with-tumor group compared with the tumor-free group. In contrast, among the TMB subgroups, low-TMB tended to have a lower risk score. However, risk scores did not differ significantly between subgroups in other clinical characteristics (age, grade, stage, and race).

To demonstrate the prognostic predictive independence of the TMB-IRS signature in multiple clinical features, we employed univariate and multivariate Cox proportional hazards regression for analysis. As shown in Table 1, univariate Cox analysis results showed that age, cancer status, TMB and OS were significantly associated with OC patients. Furthermore, multivariate regression analysis demonstrated that the TMB-IRS signature could serve as an independent predictor for evaluating the prognosis of OC patients.

**TABLE 1:** Univariate/multivariate Cox regression analysis of OC clinicopathological characteristics associated with OS.

Variables		Patient N (271)	Univariate analysis		Multivariate analysis	
			HR^a^ (95% CI^b^)	*p*	HR (95% CI)	*p*
Age	<65	95	1 (reference)	—	1 (reference)	—
	≥65	176	0.715(0.521,0.982)	0.038^c^	0.656(0.465,0.926)	0.016^c^
Stage	Stage I	18	1 (reference)	—	—	—
Stage	Stage I-II	18	1 (reference)	0.504	—	—
	Stage III	204	1.429(0.628,3.251)	0.395	—	—
	Stage IV	46	1.647(0.687,3.950)	0.263	—	—
Grade	G2	32	1 (reference)	—	—	—
	G3	229	1.001(0.636,1.574)	0.997	—	—
Cancer_status	Tumor free	71	1 (reference)	—	1 (reference)	—
	With tumor	165	8.343(4.228,16.464)	<0.001^c^	6.609(3.318,13.165)	<0.001^c^
TMB	low TMB	135	1 (reference)	—	1 (reference)	—
	high TMB	136	0.654(0.479,0.892)	0.007^c^	0.816(0.578,1.151)	0.258
TMB-IRS	—	271	1.944(1.638,2.307)	<0.001^c^	1.758(1.425,2.168)	<0.001^c^

a HR, hazard ratio.

b CI, confidence interval.

c *p* < 0.05.

## Development and Evaluation of the Nomogram

**FIGURE 4:**
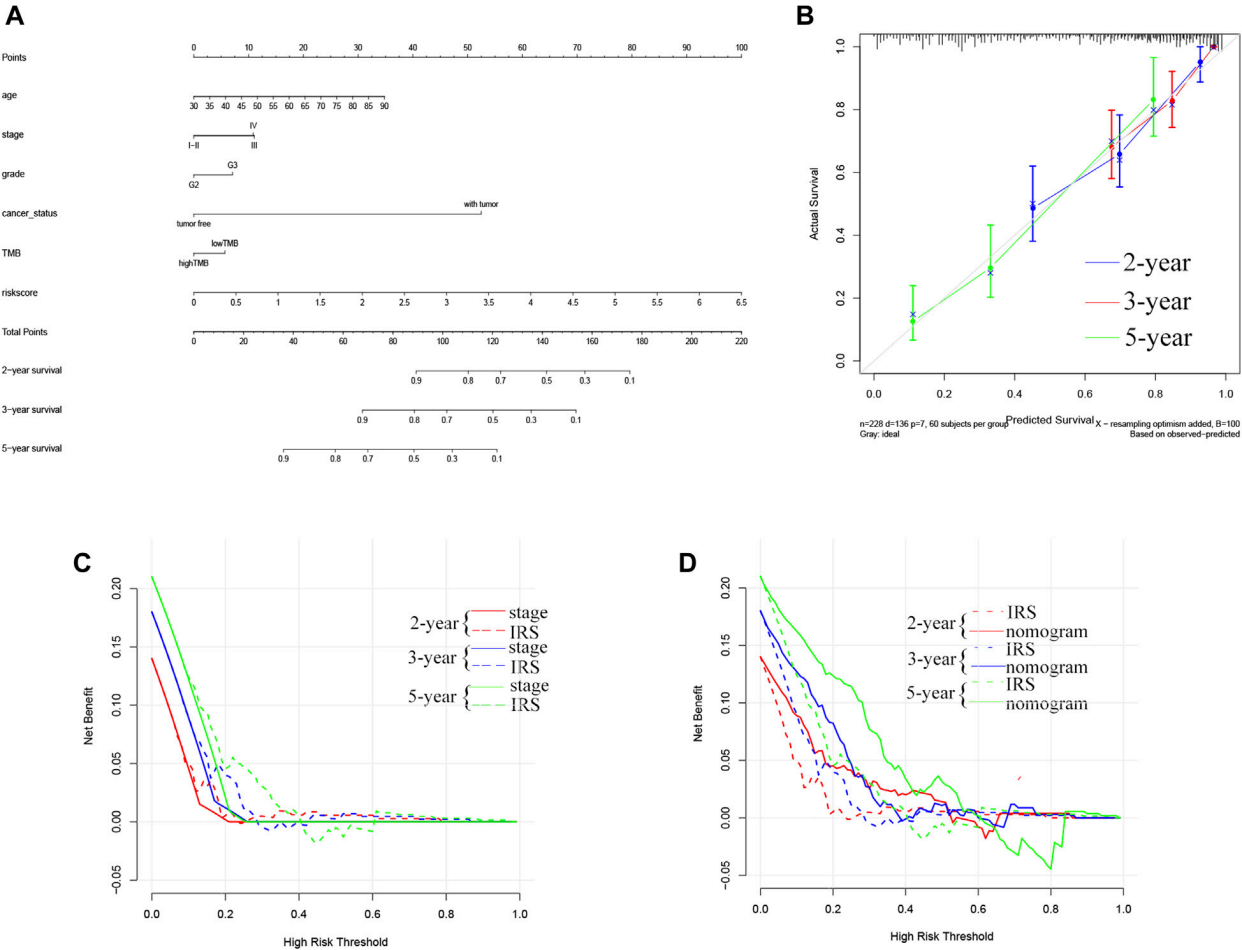
Establishment of the OS nomogram for OC patients. **(A)** Nomogram for predicting OS of OC. There are eight components in this nomogram: age, stage, grade, cancer status, and risk score. **(B)** The Calibration curve of the nomogram for predicting OS rate at 2, 3, 5 years **(C,D)** Decision curve analysis for the evaluation of the net benefits of TNM-stage, IRS and nomogram at 2, 3, 5 years.

To systematically predict the prognosis of OC, we constructed a nomogram model based on the risk score and clinical information in the TCGA dataset (Figure 4A). The calibration curve results showed that the prediction of prognostic survival probability of OC patients by the nomogram had good agreement with the actual probability (Figure 4B). Meanwhile, the C-index (95% confidence interval) of the nomogram, TNM stage, and TMB-IRS was 0.739 (0.717, 0.716), 0.643 (0.618, 0.668), and 0.537 (0.517, 0.557), respectively, and this result also demonstrated that the nomogram had better predictive accuracy. Consistent with this result, DCA plots (Figures 4C,D) also proved that TMB-IRS performed better than traditional TNM-stage for prediction, however, nomograms combining multiple clinical features had the best clinical application value.

## Tumor Immune Infiltration in OC

**FIGURE 5:**
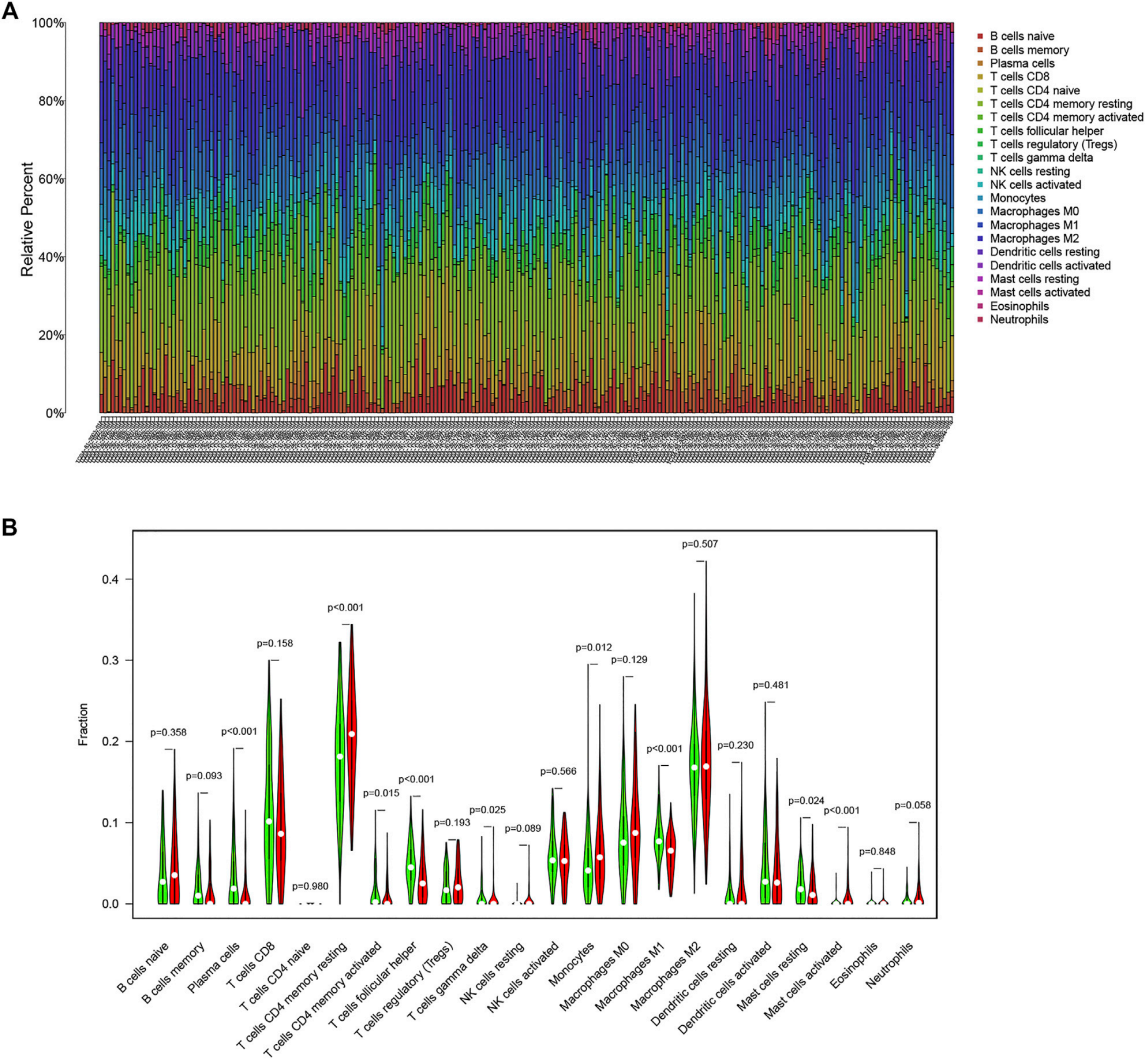
Immune infiltration in OC. **(A)** Bar plot of the proportion of 22 immune cells in the TCGA-OC patients **(B)** Comparison of 22 immune cell infiltration between the two groups in the high-risk and low-risk groups (red means the high-risk group; green means the low-risk group).

To explore the potential relationship between our risk score system and the immune infiltration microenvironment, we analyzed the correlation between the TMB-IRS and infiltrating immune cells using the “CIBERSORT” tool. The landscape of 22 immune cell infiltrates from each OC sample in TCGA was shown in Figure 5A. Figure 5B showed that Plasma cells, T cells CD4 memory activated, T cells follicular helper, Monocytes, Macrophages M1, and Mast cells resting were higher infiltrating in low-risk groups, while T cells CD4 memory resting, T cells gamma delta and Mast cells activated was higher infiltrating in high-risk groups. Supplementary Figure S1 showed that CD4 memory resting, NK cells resting, Macrophages M0, Mast cells activated and Neutrophils were positively correlated with the risk score, while Plasma cells, CD4 memory activated, T cells follicular helper, T cells gamma delta, Macrophages M1, Mast cells resting were negatively correlated with the risk score. Patients in the low-risk group had higher proportions of immune cell infiltration, with a *p* < 0.05.

## Relationship Between Immune Checkpoints and TMB-IRS

**FIGURE 6:**
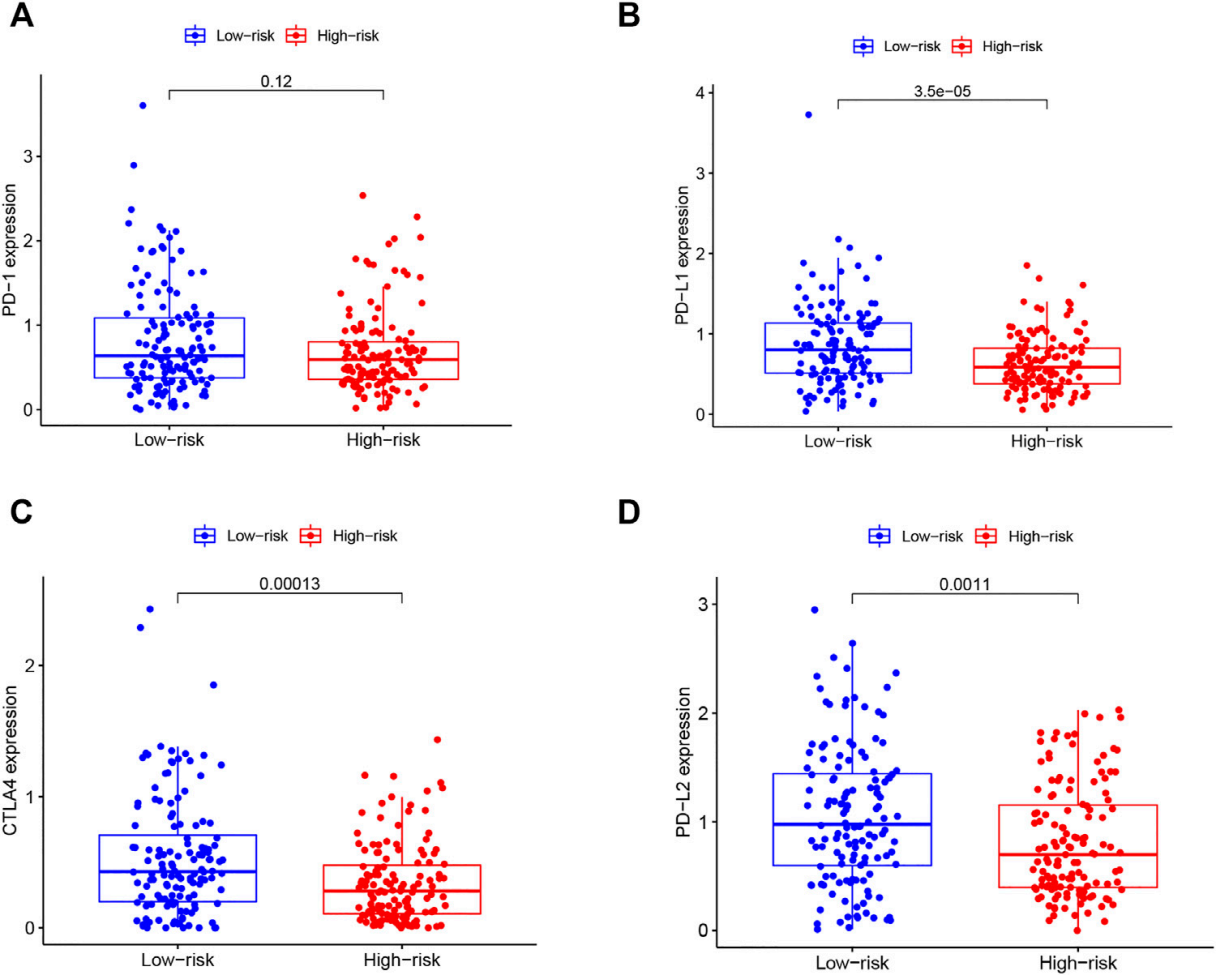
Correlation of immune checkpoint molecules with risk score **(A–D)** boxplots of PD1, PDL1, CTLA4, PD-L2 expression of OC patients in high and low-risk groups.

In recent years, cancer immunotherapy utilizing ICIs has shown promising efficacy in a proportion of cancer patients (O'Donnell et al., 2019). To explore the application value of our established TMB based model in immunotherapy, we plotted boxplots for the comparison between the expression levels of immune checkpoint molecules (PD1, PD-L1, PD-L2, CTLA4) between high-IRS and low-IRS. The results (Figures 6A–D) showed a significant negative correlation between the expression of PD-L1 (*p* < 0.001), PD-L2 (*p* = 0.001) as well as CTLA4 (*p* < 0.001) and the TMB-IRS. Specifically, the high-IRS group, with relatively lower expression of immune checkpoint genes, whereas in the low-IRS, gene expression was higher. Interestingly, there was no statistical difference in the expression of the PD-1 gene between the two groups (*p* = 0.120).

## Discussion

OC is one of the common gynecological malignancies, with 14,070 patients dying of OC in 2018 in the United States alone, and most patients are already at an advanced stage at the time of diagnosis with a poor prognosis (Torre et al., 2018). Immunotherapy has become a promising personalized therapy for OC, but there is still a lack of reliable molecular biomarkers to distinguish patients with potential sensitivity to immunotherapy (Finkelmeier et al., 2018). Therefore, it is particularly important to identify more immune-related prognostic biomarkers, which can be used as potential therapeutic targets or can be used to screen patients sensitive to immunotherapy (Odunsi, 2017). TMB is a new type of biomarker that predicts the response of cancer immunotherapy. The findings of Wang et al. indicated that high TMB could promote antigen expression and inflammatory response of testicular tumors, and patients with high TMB might achieve a better prognosis if treated with immunotherapy (Wang and Li, 2019; Yan et al., 2020). However, few studies have focused on the prognostic role of TMB and the association between TMB and OC immune cell infiltration. Therefore, in this study, we aimed to explore the prognostic role of TMB-related immune genes and their potential association with immune infiltration.

It is well known that cancer is a genetic disease and that neoplastic transformation results from the accumulation of somatic mutations in the DNA of diseased cells (Chan et al., 2019). In our study, missense mutations are the most common type of mutation in OC, and *TP53* mutations are the most frequently mutated gene, which can be identified in more than 90% of OC samples. The tumor suppressor gene *TP53* encodes the tumor suppressor protein p53, and its mutations are abundantly reported to be associated with poor prognosis in a variety of cancers (Luo et al., 2018; Li et al., 2019). In the current study, the high-TMB group has a more favorable prognosis, and conversely, the low-TMB group has a significantly poorer outcome. Yin et al.(Yin et al., 2020) suggested that high TMB could induce immune responses in humans, resulting in inhibition of tumor growth, followed by a relatively high survival rate of patients.

To screen out immune genes related to the prognosis of OC, immune-related genes were selected from the DEGs for univariate Cox and LASSO regression analysis. Nine independent prognostic immune genes associated with TMB were screened out and established a prognostic TMB-IRS signature. Among them, *DKK1*, *PI3*, *DUOX1*, *PAEP* and *CXCL10* genes are positively correlated with OS, while *CSPG5*, *CXCL11*, *TNFRSF17* and *TNFRSF13B* genes are negatively correlated with OS. Chondroitin sulfate proteoglycan 5 (*CSPG5*) encodes human chondroitin GSPG5, which is related to immune-related genes that are prognostic indicators of breast cancer and liver cancer patients (Shi et al., 2020). *CXCL10* and *CXCL11* are ligands of chemokine CXCR3, which can regulate the migration, differentiation and activation of immune cells, and are related to the selective migration and linear development of CD4 + and CD8 + T cells (Karin and Razon, 2018), thereby affecting the therapeutic effect of cancer (Tokunaga et al., 2018). DKK1, a regulator of Wnt signaling, is found to affect the tumor microenvironment by suppressing tumor immunity and can be used as an immunotherapeutic target for OC (Betella et al., 2020), which is consistent with our research results. *TNFRSF17* and *TNFRSF13B*, members of the tumor necrosis factor receptor superfamily, are primarily involved in the maturation of B lymphocytes and are associated with tumor growth and invasiveness and may serve as therapeutic targets in breast cancer (Pelekanou et al., 2018). Previous studies have shown that dual oxidase 1 (DUOX1) is commonly downregulated in lung, liver, and breast cancers, suggesting that it may have a tumor suppressor role (Little et al., 2016; Fortunato et al., 2018). Progesterone-associated endometrial protein (PAEP) can be used as a non-invasive biomarker to break down endometriosis (Irungu et al., 2019). Studies have reported its utility as a biomarker and immune system modulator in non-small cell lung cancer (Weber et al., 2019) and its association with prognosis in bladder cancer (Liu L et al., 2020). However, the prognostic relevance of PI3 in cancer has been less frequently reported, which may shed light on the mechanistic investigation of a novel immune gene in cancer.

In our study, KM analysis and ROC curve results confirm the favorable prognostic predictive value and accuracy of our established TMB-IRS signature. Specifically, the TMB-IRS signature can stratify patients into high- and low-risk groups with different outcomes and immunophenotypes, and the high-risk group is significantly associated with poor prognosis. Further, we have determined the relationship between the established model and multiple clinicopathological factors (age, cancer status, grade, stage, and ethnicity). TMB-IRS is significantly positively correlated with cancer status but negatively correlated with TMB, which is consistent with previous studies (Chan et al., 2019). Univariate and multivariate Cox regression results indicate that TMB-IRS, tumor status and age are independent prognostic predictors for the prognosis of OC patients. To comprehensively evaluate the prognosis of patients, we also establish a novel comprehensive nomogram risk assessment model based on clinical information. DCA and C-index results show that the predictive accuracy of TMB-IRS is higher than traditional TNM staging, while the nomogram containing multiple clinical information has the best prognostic predictive accuracy.

Accumulating evidence suggests that the immune component of the TME may be highly involved in tumor progression, as an immunosuppressive TME is associated with a worse patient prognosis (Tsogas et al., 2021). Immune cell infiltration in the tumor microenvironment can affect the treatment response and outcome of OC (Chalmers et al., 2017). Our research results show that Plasma cells, T cells CD4 memory activated, T cells follicular helper (Tfh), Monocytes, Macrophages M1, and Mast cells resting are higher infiltrating in low-risk groups, while T cells CD4 memory resting, T cells gamma delta and Mast cells activated is higher infiltrating in high-risk groups. This indirectly proves that the high immune response can inhibit the growth of OC tumors and improve the prognosis. Hollern et al. found that immune checkpoint therapy could induce the activation of Tfh of B cells, thereby promoting the anti-tumor response in a mouse model of triple-negative breast cancer (Hollern et al., 2019). In this study, 12 cells out of 22 immune cells were significantly correlated with TMB-IRS, and three of these cells (macrophage M1 T cell follicular helper plasma cells) were highly correlated with TMB-IRS (R > 0.3). High-affinity antibodies secreted by B cells and plasma cells are essential for the organism to fight and clear pathogen infections, whereas germinal center formation, B cell differentiation, and antibody affinity maturation are all independent of follicular helper T cell help, and macrophage M1, a macrophage that can produce proinflammatory cytokines, has strong microbial killing properties (He et al., 2018). In our study, the lower these three cell levels were when TMB-IRS was higher, which explained the potentially threatening and poor prognosis of tumors to some extent.

Currently, to effectively predict the prognosis of tumor patients, a large number of models matching the prognosis of tumor patients have been established and validated. For example, Shen et al. developed a promising biomarker based on immune genes that could predict overall survival in OC through the Immport database (Shen et al., 2019). Using a TMB-associated signature to predict OS in OC, Bi et al. concluded that TMBB plays a critical role in the prognosis of OC and guides immunotherapy (Bi et al., 2020). In the study of fan et al.(Fan et al., 2020), the TMB-related genes were obtained by constructing the WGCNA network, and we were the DEGs obtained by differential analysis. Liu et al.’s (Liu J et al., 2020) study constructed a prognostic risk score for EOC (epithelial ovarian cancer) by obtaining all genes associated with TMB, while our study focused on the prognostic predictive role played by immune genes in OC. However, in our study, based on TMB high and low grouping, a signature constituted by 9 immune genes was established, which could more accurately predict the prognosis of OC, suggesting the level of immune cell infiltration, and thus guide immunotherapy.

ICIs with blocking antibodies targeting cytotoxic T lymphocyte antigen-4 (CTLA-4) as well as the programmed cell death protein 1 (PD-1) pathway and programmed death-1/programmed death-ligand 1 (PD-L1) has shown promising results in a variety of malignancies including OC (Odunsi, 2017; Memon and Patel, 2019). In our study, the expression of these immune checkpoint molecules was inversely correlated with that of TMB-IRS, suggesting a potential predictive role of our model for individual response to immunotherapy.

In the current study, we first explore the correlation between TMB and the prognosis of OC, and the results show that higher TMB levels are significantly associated with a better prognosis of OC. Based on the TMB score, nine TMB associated immune genes are identified, from which a biomarker TMB-IRS is constructed that can also effectively predict the prognosis of OC. We find that the TMB-IRS signature is negatively correlated with infiltrating immune cells, a new robust TMB-IRS signature, to help clinicians determine the most likely benefit from immunotherapy. The TMB-IRS signature, based on its strong prognostic predictive value and its association with immunotherapy, may serve as a novel biomarker and potential therapeutic target for predicting OC prognosis. The present study is a retrospective study, which is a limitation, so further prospective studies and clinical validation of its analytical accuracy and testing its clinical utility are warranted.

## Data Availability

The original contributions presented in the study are included in the article/Supplementary Material, further inquiries can be directed to the corresponding authors.
