# Supplementary figures and images for: Development and Validation of a Tumor Mutation Burden-Related Immune Prognostic Signature for Ovarian Cancers
Source: Front Genet. 2022 Jan 11;12:688207. doi: 10.3389/fgene.2021.688207 (PMC8787320; doi:10.3389/fgene.2021.688207)

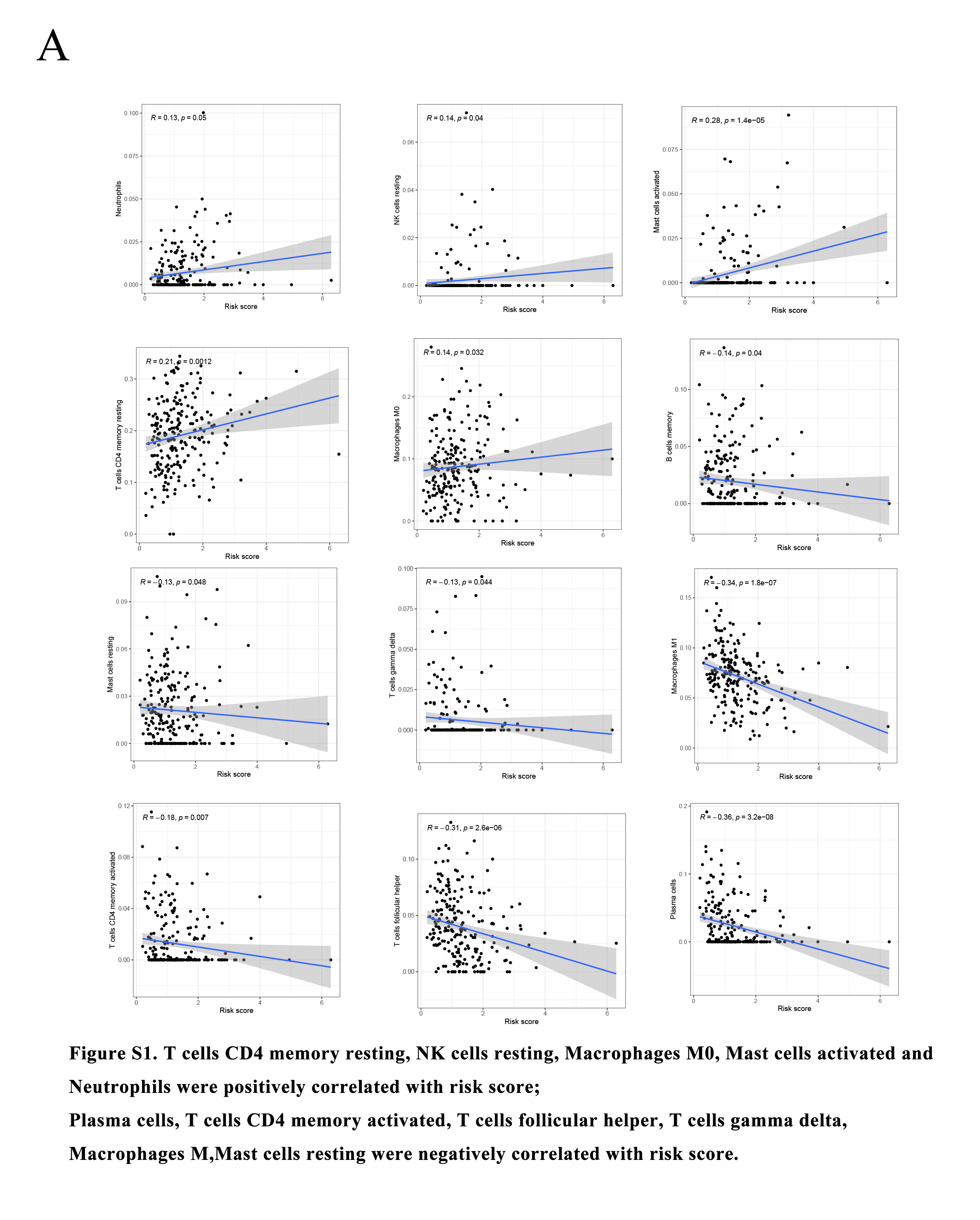

Supplement: Supplementary file 1 [file Image1.TIF]
